# 2021: New horizons in neurorehabilitation

**DOI:** 10.25122/jml-2021-1004

**Published:** 2021

**Authors:** Dafin Muresanu

**Affiliations:** 1. Iuliu Hatieganu University of Medicine and Pharmacy, Cluj-Napoca, Romania; 2. RoNeuro Institute for Neurological Research and Diagnostic, Cluj-Napoca, Romania

Difficult times ask for innovative and daring solutions to complex issues. In the interest of the health, safety, and well-being of all registered attendees and the general public, the Society for the Study of Neuroprotection and Neuroplasticity (SSNN) Board of Directors has decided to extend last year’s decision regarding all upcoming events and hold the conferences in a virtual environment. We are committed to supporting public health authorities globally in their effort to slow and contain the spread of COVID-19. We must stay positive, healthy, and united more than ever to overcome this crisis.

Our well-established conferences – the 11^th^ European Teaching Course on Neurorehabilitation (ETCN) (17–18 September 2021) and the 16^th^ International Summer School of Neurology (19–21 September 2020) will be offering outstanding opportunities for education, dissemination of scientific research, and the exchange of best practices, through a brand new online platform that has been tailored to promote lively interaction between participants.

The current virtual events benefit from the active support of some of the longstanding partners like the European Federation of Neurorehabilitation Societies (EFNR), World Federation of Neurorehabilitation Societies (WFNR), the European Academy of Neurology (EAN), Iuliu Hatieganu University of Medicine and Pharmacy from Cluj-Napoca, Romania, the RoNeuro Institute for Neurological Research and Diagnostic, and the Journal of Medicine and Life.

## 11^th^ European Teaching Course on Neurorehabilitation

The ETCN is one of the most acclaimed events organized by the SSNN. This year’s second online edition, out of eleven so far, promises to rise to participants’ expectations and serve as a platform for disseminating and exchanging up-to-date scientific knowledge on neurorehabilitation and providing a space for teaching-oriented workshops. Each year, the event reaches a broader audience interested in this steadily expanding and exciting field (i.e., physicians, nurses, therapists, public health professionals, and more).

The event’s focus is on rehabilitation and neurorecovery, along with identifying new avenues in science, education and delivery of service – the Scientific Program Committee, conducted by Volker Hömberg President-Elect of the World Federation Neurorehabilitation (WFNR) and Vice-President European Federation of Neurorehabilitation Societies (EFNRS), carefully curated the program developed on submissions from our top-grade international faculty.

The event’s objectives are (1) to further the development and improve quality of neurorehabilitation in Europe, (2) to enhance collaboration in neurorehabilitation between professionals, (3) to encourage and facilitate the exchange of knowledge and scientific research in rehabilitation professionals, and (4) to contribute to the development of cooperation and communication networks between national and international neurorehabilitation societies.

## 16^th^ International Summer School of Neurology

The International Summer School aims to facilitate the interaction between young neurologists-in-training and an internationally established faculty with extensive expertise in basic and clinical neuroscience. The pillars for this event were set in 2005 together with professors Natan Bornstein (Israel) and Ovidiu Bajenaru (Romania) to fulfill the need of young specialists and practitioners for connection with the latest developments in the field of neurosciences. This pursuit led to the development of a dynamic environment that promotes connection and learning. In the 16^th^ edition, coordinated by Natan M. Bornstein and Dafin F. Muresanu, the virtual event will address topics such as the COVID-19 impact, challenges of evidence-based medicine, stroke recovery monitoring, the right of patients for the suitable device aided therapies, and cost-effectiveness of interventions for neurorecovery after acute ischemic stroke.

## This year’s exciting new avenue: Guideline EAN-EFNR

The 2020 SSNN virtual events will set the stage for five days of intensive talks and debates between over a thousand participants from 25 countries on a broad range of problems in neurosciences. An exciting achievement of this year is represented by the guideline on pharmacological support in early motor rehabilitation after acute ischaemic stroke. The guideline aims to support the clinical decision-making of healthcare professionals involved in the recovery of stroke patients. They were developed using the Grading of Recommendations, Assessment and Evaluation (GRADE) framework to identify early pharmacological interventions for stroke rehabilitation delivered alongside neurorehabilitation. This guideline provides clinicians with information regarding existent pharmacological support in post-acute stroke neurorecovery intervention, highlighting an exciting avenue for research and development.

Despite the societal changes caused by the ongoing global pandemic, we are enthusiastic about finding innovative pathways of adapting to the current context. In our pursuit, we ought to work together to overcome these trying times and build a brighter future.

